# Laparoscopic management of 139 ovarian mature cystic teratomas with an emblematic case report: a single-center experience

**DOI:** 10.1097/RC9.0000000000000131

**Published:** 2026-02-13

**Authors:** Antonio Maccio, Manuela Neri, Valerio Vallerino, Gabriele Sole, Elisabetta Pusceddu, Paolo Albino Ferrari

**Affiliations:** a Department of Obstetrics and Gynecology and Gynecological Oncology, ARNAS G. Brotzu, Piazzale A. Ricchi, Cagliari, Italy; b Department of Surgical Sciences, University of Cagliari, Monserrato, Italy; c Department of Oncological Surgery, ARNAS G. Brotzu, Piazzale A. Ricchi, Cagliari, Italy; d Anesthesia and Intensive Care Unit, Liver Transplantation Center, ARNAS G. Brotzu, Piazzale A. Ricchi, Cagliari, Italy; e Division of Thoracic Surgery, Oncology Hospital A. Businco, ARNAS G. Brotzu, Cagliari, Italy

**Keywords:** case series, dermoid ovarian cyst, laparoscopy, mature cystic teratoma, squamous cell carcinoma

## Abstract

**Introduction:**

The optimal surgical route for large or potentially malignant ovarian mature cystic teratomas (MCTs) remains debated.

**Case presentation:**

We retrospectively included 139 consecutive patients with pathologically confirmed MCT managed laparoscopically at a tertiary center (June 2013–August 2024). Mean cyst size was 9 cm (range 4–20); adnexal torsion occurred in 6%. All procedures were completed minimally invasively; cystectomy and adnexectomy were performed in 40% and 60%, respectively. Intraoperative spillage was 2.2%, with no intra- or postoperative complications. One illustrative case is described: the laparoscopic removal of a giant 8.5-kg MCT and its management.

**Discussion:**

With strict containment (controlled decompression, systematic endobagging, sealed extraction) and appropriate expertise, laparoscopy is feasible and oncologically prudent even for giant MCTs and in scenarios at risk of malignant degeneration.

**Conclusion:**

In a high-expertise setting using strict no-rupture retrieval protocols, laparoscopy was feasible for MCTs across a broad size range. These results apply to benign-appearing lesions in our single-center series; the accompanying giant cyst is presented as an illustrative case and is not included in pooled outcomes.

## Introduction

Dermoid ovarian cysts are mature cystic teratomas (MCTs) that affect reproductive-age women and account for 10–20% of ovarian tumors; malignant transformation occurs in ~1–3%^[^1,2^]^. Laparoscopy is widely adopted for MCTs, offering less pain, shorter hospitalization, and better cosmesis than laparotomy^[^3,4^]^. Management of huge masses remains debated because of limited working space and rupture risk; spillage of dermoid contents can cause chemical peritonitis, adhesions, infertility, or recurrence, and, when degeneration is present, may facilitate intraperitoneal cancer spread^[^5^]^. Route selection should incorporate malignancy risk, patient factors, and technical complexity while prioritizing oncologic safety with strict containment. Against this background, we reviewed consecutive patients undergoing laparoscopic surgery for ovarian MCTs at our tertiary unit and present an emblematic extreme: the laparoscopic removal of a giant 8.5-kg MCT and its management.

**HIGHLIGHTS**

- Single-center laparoscopy series of 139 ovarian MCTs, 100% completed.
- Systematic endobag use and controlled decompression minimized spillage (2.2%).
- No intra- or postoperative complications across the entire cohort.
- Laparoscopic removal of a giant 8.5-kg MCT achieved without spillage.

## Materials & methods

We conducted a retrospective single-center case series at a tertiary referral Gynecologic Oncology Unit (June 2013–August 2024). All operations were performed by one experienced team led by a senior surgeon dual-trained in Obstetrics, Gynecology, and Medical Oncology.

Consecutive patients referred for laparoscopic management of ovarian cysts with high surgical complexity (future fertility, large size, risk of intraoperative MCT spillage and adhesions formation, or elevated CA19.9/CA125 suggesting malignancy) were screened. Only patients with histologically confirmed MCT were included. The emblematic “giant” MCT is presented as a separate case report and excluded from cohort statistics.

The study complied with the Declaration of Helsinki and received IRB approval; all patients provided informed consent for treatment and the anonymized use of their data.

Variables included demographics, cyst characteristics (radiologic size and torsion), surgical approach, spillage, and complications. Primary outcomes were the feasibility of laparoscopic completion (conversion rate) and intraoperative spillage (any macroscopic dermoid content identified outside the retrieval bag). Secondary outcomes included operative time, estimated blood loss, transfusion, postoperative complications according to Clavien–Dindo classification, and length of stay. Complications were recorded during index admission and at scheduled follow-up visits.

Procedures were laparoscopic cystectomy or adnexectomy according to reproductive goals, cyst size, tissue quality, and intraoperative findings, using a systematic endobag strategy for specimen containment. For very large cysts, controlled decompression and sealed extraction were employed to minimize spillage, with reinsertion of the cyst into the retrieval bag prior to definitive removal when decompression was needed. Conversion to laparotomy was reserved for findings incompatible with safe minimally invasive completion^[^6^]^.

All patients underwent preoperative ultrasonography (transvaginal and/or transabdominal) described using International Ovarian Tumor Analysis (IOTA) terminology (e.g., dermoid mesh, Rokitansky nodule, echogenic sebaceous material, acoustic shadowing). When sonographic features were typical for MCT and lacked malignant stigmata (e.g., solid papillary projections with vascular flow, ascites), patients proceeded to laparoscopy. For indeterminate or very large lesions, or when alternative pathology was suspected, computed tomography (CT) was obtained to corroborate fat–fluid levels, calcifications, and mural nodules. Tumor markers (e.g., CA125, CA19-9; HE4 when available) complemented imaging; isolated marker elevations without corroborating imaging did not preclude laparoscopy when the overall assessment favored benign MCT. Patients with clinical/imaging concern for malignancy were triaged to a staging-capable pathway. Suspected torsion prompted urgent laparoscopy. This integrated pathway underpinned the selection of a minimally invasive approach with strict containment/no-spillage precautions in all cases.

In-hospital monitoring included daily clinical assessments and laboratory tests (e.g., C-reactive protein, fibrinogen). After discharge, planned follow-up occurred at 1 week, then every 4 weeks for the first 3 months, and subsequently at 6 and 12 months, with earlier evaluation if clinically indicated. Recurrences, readmissions, and late complications were captured during visits and by review of the institutional electronic record.

Data were abstracted from the institutional electronic medical record into a secure database. Continuous variables are summarized as mean ± SD or median (range); categorical variables as counts (percentages). No imputation was performed for missing data; analyses were descriptive, consistent with the observational design.

This study was reported in line with the SCARE criteria^[^7^]^.

## Institutional report

From June 2013 to August 2024, we included 139 consecutive patients with ovarian MCT. Median age was 48 years (range, 19–68). Mean cyst size was 9 cm (SD ± 3, range 4–20). Eight patients (6%) presented with adnexal torsion. All procedures were performed using minimally invasive techniques (100%). Ovarian cystectomy was performed in 56 cases (40%) and adnexectomy in 83 (60%). The intraoperative spillage rate was 2.2% (3/139). No intraoperative or postoperative complications were recorded according to Clavien–Dindo classification. Table 1 reports the main characteristics of MCTs and the surgical procedures performed in this cohort. Beyond the series, we described an emblematic report (Case 1) with image and surgical video: the laparoscopic removal of a giant 8.5-kg MCT in a postmenopausal patient.

**Table 1:** Patients’ clinical characteristics and operative data of ovarian MCTs

Parameter	
Patients enrolled, no.	139
Age, years: median (range)	48 (19–68)
Height, cm: median (range)	168 (158–173)
Weight, kg: median (range)	68 (50–89)
BMI: median (range)	23.5 (19–29)
Clinical signs	
Abdominal/pelvic pain, No. (%)	33 (23.7)
Abdominal mass, No. (%)	13 (9.4)
Constipation, No. (%)	2 (1.4)
Size, cm: mean ± SD (range)	9 ± 3 (4–20)
Torsion: No. (%)	8 (6)
Surgical procedure: No. (%)	
Cystectomy	56 (40)
Adnexectomy	83 (60)
Rate of spillage: No. (%)	3 (2.2)
Intraoperative/postoperative Complications	0
Surgical time, minutes: median (range)	53 (30–120)
Blood loss, mL: median (range)	150 (0–500)
Conversion to laparotomy, No.	0

No.: number, cm: centimeters, kg: kilograms, BMI: body mass index, SD: standard deviation, mL: milliliters

## Case 1

**Figure 1.**
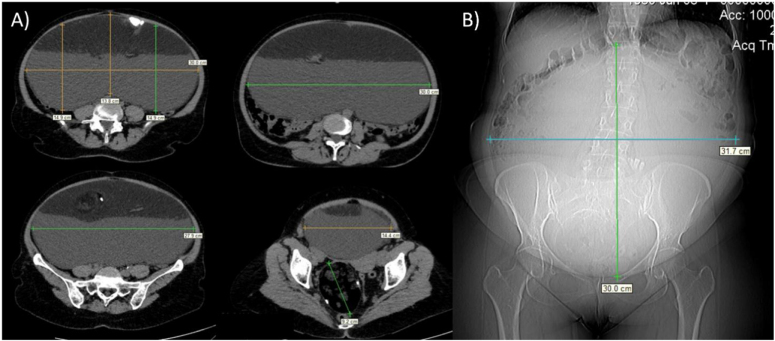
CT scan images of Case 1. (A) Abdominal transverse CT scan showed the left cyst measuring 317 × 300 mm with its liquid component and the right cyst measuring approximately 90 × 50 mm; (B) Longitudinal scan showed the hugest cyst measuring 317 × 300 mm extending from the pelvis to the mesogastrium.

A 60-year-old Caucasian woman was admitted to the Department of Gynecologic Oncology with a 12-month history of vague abdominal pain and swelling that had progressed over the prior 4 months to persistent abdominal pain, constipation, and mild dyspnea. She had no previous illnesses and no family history of cancer. On examination, a pelvic–abdominal mass caused marked distension from the pubic symphysis to ~5–6 cm above the transverse umbilical line; body mass index was 21 kg/m^2^. Serum tumor markers (CA125, carcinoembryonic antigen, CA19-9, CA15-3, and HE4) were within normal limits. Transabdominal ultrasonography showed a >30 cm cystic lesion filling the abdomen with mixed echogenicity, dermoid mesh, and Rokitansky nodules, without papillary projections or increased intralesional blood flow; no ascites was detected, suggestive of a giant ovarian MCT^[^8^]^. CT revealed a large left-sided cyst extending from the pelvis to the mesogastrium and a smaller right pelvic cyst; both contained greasy fluid, calcifications, and soft-tissue elements. The left cyst measured 317 × 300 mm and the right ~ 90 × 50 mm, consistent with bilateral ovarian MCT (Fig. 1).

After counseling, a laparoscopic approach was chosen as the most efficient management strategy. The patient consented to laparoscopic bilateral salpingo-oophorectomy, total hysterectomy, omentectomy, and conversion to laparotomy if needed. The case was conducted in accordance with the Institutional Ethics Committee and the Helsinki Declaration; written informed consent for publication of the report and images was obtained.

Under general anesthesia in lithotomy, controlled decompression was first performed to minimize spillage. A ~1.5 cm infraumbilical incision was carried down to the cyst wall; a 14-G Venopic AGO cannula was used to puncture the cyst, and ~400 mL of corpuscular fluid were aspirated with a 50/60 mL Luer-lock syringe, achieving partial decompression^[^9^]^. The cyst wall was then elevated with two thin forceps to permit a small controlled incision and insertion of a laparoscopic suction cannula connected to a Hamou Endomat (Karl Storz). An additional 3.2 L of yellow, corpuscular fluid were evacuated.

Laparoscopic inspection demonstrated a large, decompressed left ovarian cyst with smooth, intact wall; the liver, bowel, and diaphragm were normal, without macroscopic malignancy. The uterus was regular and normal-sized. Using a LigaSure™ Blunt Tip device (Covidien/Medtronic), a right salpingo-oophorectomy was undertaken, during which a ~10 cm right ovarian cyst was identified. The right MCT was secured in a 15 cm detachable endo pocket (ConMed), aspirated, and removed. Left salpingo-oophorectomy followed with coagulation and transection of the left round, utero-ovarian, and infundibulopelvic ligaments using the same device. A total laparoscopic hysterectomy was then performed according to our standard technique^[^10^]^. The giant left cyst was placed in a 50 cm 3 M Steri-Drape™ isolation bag and gradually extracted transvaginally with external aspiration of sebaceous contents, achieving complete removal without tumor spillage. Approximately 4 L of sebaceous fluid were drained; the cyst wall weighed ~900 g, for a total MCT weight >8.5 kg. Operative time was ~120 minutes, with no blood loss and no intraoperative or anesthetic complications. The full laparoscopic procedure is presented in Supplementary Video (https://doi.org/10.5281/zenodo.13834903).

Histopathologic examination confirmed a mature teratoma without cytologic atypia. The postoperative course was uneventful; the patient was discharged on postoperative Day 2 and promptly resumed normal daily activities, remaining in good health at 3 years follow-up.

## Discussion

In our series, laparoscopic removal of ovarian MCTs using a retrieval-bag protocol was feasible with high completion (139/139) and low spillage (3/139), with no adjudicated perioperative complications during structured follow-up (95% CI for spillage ~0.7–6.2%). These outcomes compare favorably with published laparoscopic cohorts and are likely due to standardized containment and team experience.

A brief quantitative comparison with larger series supports the generalizability of our findings. Sinha *et al*^[^3^]^ pooled 655 laparoscopic and 409 open procedures for ovarian mature cystic teratoma, showing higher rates of cyst content spillage with laparoscopy but extremely rare chemical peritonitis when meticulous lavage is performed. Lee *et al*^[^11^]^ reported 132 surgically treated OMCTs, with adnexal torsion in 37 cases (28%), laparoscopic access in about 70–80% of patients, and cystectomy favored over oophorectomy. Our complication profile and absence of chemical peritonitis therefore align with these larger experiences, despite the inclusion of giant lesions.

Critical technical steps for giant lesions include controlled periumbilical decompression using needles of different gauges, sealed reinsertion of the cyst, systematic bagging, meticulous aspiration of any escaped contents, and transvaginal extraction of the bag-contained mass—yielding oncologic safety and excellent cosmetic recovery. Various adjuncts have been proposed to mitigate rupture—preventive laparoscopic inspection, minilaparotomy support, LapDisc use, and routine endobagging—though robust comparative data remain limited^[^5,12,13^]^. If rupture occurs, extensive waterjet irrigation has been described as a preventive measure against complications, yet it is neither fully reliable nor time-efficient in practice^[^14,15^]^. Our approach aligns with isolated reports of laparoscopic management of giant dermoid cysts after controlled decompression, reinforcing the technical plausibility of a minimally invasive route even at extreme sizes^[^16,17^]^. Technique selection must also consider the possibility of malignant degeneration; when present, intraperitoneal dissemination after cyst rupture has been reported, underscoring strict adherence to containment and no-spillage principles^[^5,18^]^.

This retrospective, single-center experience (2013–2024) reflects an expert tertiary pathway and may not fully generalize to lower-volume settings. Temporal changes in imaging, instrumentation, and care pathways introduce potential confounding. Outcome capture relied on institutional records, so rare or external events may have been missed; patient-reported outcomes were not systematically collected. In addition, we did not prospectively collect structured long-term fertility data (e.g., attempts at conception, pregnancy rates) after surgery, and follow-up for late recurrence beyond the first postoperative year was not uniform, which limits our ability to draw firm conclusions about reproductive outcomes and long-term recurrence risk. Spillage was recorded macroscopically rather than cytologically. The study was not powered for malignant transformation or oncologic endpoints, warranting prospective multicenter confirmation.

Our recommendation to prioritize laparoscopy pertains to adnexal masses that remain convincingly benign-appearing after preoperative assessment. When preoperative or intraoperative cues raise credible concern for malignancy, operative goals shift: laparoscopy is pursued only if oncologic endpoints can be safeguarded—no rupture, in-bag retrieval, and en-bloc adnexectomy when indicated—with early conversion or primary staging if those conditions cannot be assured. A selective frozen section may assist decisions where available. These guardrails delineate the threshold beyond which minimally invasive surgery should yield to oncologic imperatives, ensuring that feasibility is never prioritized over oncologic integrity. Indeed, the need to refer suspected large, complex, or potentially malignant MCTs to high-expertise centers: suboptimal preparation, technique, or experience can precipitate life-threatening sequelae.

## Conclusion

The upper size limit for a laparoscopic approach should not be viewed as absolute. With diagnostic laparoscopy for operative planning, strict containment strategies (decompression, bagging, controlled extraction), and an experienced multidisciplinary team, laparoscopy can be prioritized without compromising oncologic safety -even in giant MCTs. Referral to high-expertise centers is advisable; ultimately, surgeon competence, not tumor size alone, should drive the choice between laparoscopy and laparotomy.

## Data Availability

Retrospective anonymized data were collected on all consecutive cases and held locally in a secure database at the Department of Gynecologic Oncology, and are available on request from the corresponding author.
